# Real-time tracking of *Yersinia pestis* infection dynamics using a near-infrared fluorescent protein labeling system

**DOI:** 10.1128/spectrum.00959-26

**Published:** 2026-09-10

**Authors:** Yiqian Wang, Tong Wang, Hongyan Chen, Yu Zhang, Jiaxing An, Zehui Tong, Yajun Song, Dianbing Wang, Ruifu Yang, Yafang Tan, Zongmin Du

**Affiliations:** 1 State Key Laboratory of Pathogen and Biosecurity, Academy of Military Medical Sciences 71040, Beijing, China; 2 Center for Infection Biology, School of Basic Medical Sciences, Tsinghua University 12442, Beijing, China; 3 Jiangsu Key Laboratory of Zoonosis/Jiangsu Co-Innovation Center for Prevention and Control of Important Animal Infectious Diseases and Zoonoses, Yangzhou University 38043, Yangzhou, China; 4 Institute of Biophysics, Chinese Academy of Sciences 53015, Beijing, China; Mercy LLC, Roswell, Georgia, USA

**Keywords:** near-infrared fluorescence, *in vivo *imaging, *Yersinia pestis*, host cell-pathogen interactions, infection dynamics

## Abstract

Live imaging is essential for elucidating dynamic infection processes. Here, we developed a near-infrared fluorescent protein (NIR-FP)-based labeling strategy that enables integrated visualization of bacterial behavior in both cellular and animal infection models. Using *Yersinia pestis* as a model pathogen, we constructed stable fluorescent strains by either chromosomal insertion of the gene encoding NIR-FP miRFP670nano3 under the control of a strong endogenous promoter to obtain Yp-nano3, or by introducing a plasmid expressing the same reporter to obtain Yp-p-nano3. Both labeled strains exhibited a strong correlation between fluorescence intensity and bacterial counts, with Yp-nano3 exhibiting significantly higher brightness at the same bacterial count. Importantly, the labeled strains showed no measurable reduction in virulence under the tested experimental conditions, supporting their use in physiologically relevant infection studies. In cellular infection assays, sustained and robust fluorescence from the labeled bacteria enables continuous monitoring of pathogen–host cell interactions for up to 12 h. In a murine bubonic plague model, fluorescence signals were detectable in the inguinal lymph nodes (ILN) as early as 1 day post-infection with Yp-nano3 and increased progressively until the mice died. In contrast, mice infected with a highly attenuated *Δpla* mutant carrying the same NIR-FP label showed no detectable fluorescence in ILN throughout the infection. These results show that NIR-FP labeling, combined with *in vivo* imaging, provides a powerful approach for real-time tracking of the dissemination of *Y. pestis* in lymph nodes and for assessing bacterial virulence. Together, this work establishes an NIR-FP labeling platform suitable for real-time analysis of bacterial infection dynamics and host-pathogen interactions.

**IMPORTANCE:**

Live imaging techniques, such as bioluminescence imaging and fluorescence imaging, have been widely used to study infection dynamics. However, bioluminescence imaging is strongly limited by its dependence on exogenous substrate administration and its inability to explore direct interactions between pathogen and host cells. To overcome these limitations, we established an NIR-FP labeling platform that enables visualization of pathogen–host interactions at both cellular and animal levels, using *Y. pestis* as a model pathogen. This labeling method offers high stability and a strong linear relationship between fluorescence intensity and bacterial count, allowing real-time tracking of bacterial dissemination to lymph nodes and subsequent proliferation *in vivo*. Together, these attributes offer a novel and robust tool for investigating infection dynamics and underlying pathogenic mechanisms.

## INTRODUCTION

Pathogens such as *Yersinia pestis* (1, 2), *Staphylococcus aureus* (3, 4), and *Streptococcus pyogenes* (5, 6) initiate subcutaneous infections through mechanical injury or vector-borne transmission, subsequently progressing to systemic infection via the lymphatic circulation. The pathogens’ dissemination to the lymph nodes, replication, and colonization thereafter are critical for the severity of infections and the disease outcome. Therefore, investigation of the dynamic progress of these infections, particularly the transmission of the bacterial pathogens to the lymph nodes, often provides crucial insights into pathogenic mechanisms.

Conventional methods for assessing dynamic infection processes are labor-intensive and time-consuming, typically involving the measurement of bacterial burden in tissues from animals sacrificed at different time points (7, 8). This endpoint-based approach necessitates the use of multiple animal groups for different time points to compensate for the inability to track bacterial dissemination in the same subject throughout infection. Difficulty in conducting such labor-intensive time-series studies strongly limited its ability to fully capture the details of infection progression. Recent advancements of live imaging technology allow for continuous *in vivo* monitoring of the real-time dynamics of infection (9–11).

To visualize the dynamic progression of infection *in vivo*, pathogens labeled with different optical reporters have been widely used to infect animals, enabling monitoring of infection through *in vivo* imaging. According to the mechanism of light emission, these live imaging approaches are primarily classified into bioluminescence imaging and fluorescence imaging (12). In bioluminescence imaging, light is produced through enzymatic reactions involving specific luciferases and their corresponding substrates (13). These substrates can be catalyzed by their respective co-luciferases, resulting in the emission of visible light. Unlike bioluminescence imaging, fluorescence imaging relies on small-molecule fluorescent dyes or fluorescent proteins. These molecules absorb light at specific excitation wavelengths, enter an excited state, and subsequently emit light as they return to their ground state.

While fluorescent proteins such as GFP and RFP are widely used in molecular biology (14, 15), their application in live imaging is hindered by limited tissue penetration and the interference of autofluorescence (16, 17). Near-infrared (NIR) fluorescence, characterized by its longer emission wavelength, presents distinct advantages for live imaging, including lower tissue absorption, reduced scattering, and deeper penetration (18–20). Nevertheless, studies employing NIR fluorescent protein labeling for dynamic visualization of bacterial infection remain limited.

In this study, we developed two novel labeling methods by integrating a gene encoding NIR fluorescent protein miRFP670nano3 into *Y. pestis* chromosome or by introducing a miRFP670nano3-expressing plasmid. These innovative methods enabled real-time visualization of the dynamic infection process, both in cell infection assay and in a murine model of bubonic plague. Additionally, we demonstrate that the established methods can be used to assess the virulence of different *Y. pestis* mutants. Our approach offers a reliable method for tracking bacterial infections, thus providing powerful tools for the research of infectious diseases.

## RESULTS

### Establishment of miRFP670nano3 labeling method for *Y. pestis*

miRFP670nano3 (21) is a single-domain NIR fluorescent protein derived from cyanobacteriochromes (CBCRs), exhibiting fluorescence excitation at 645 nm and emission at 670 nm. This protein is characterized by its small size, high brightness, and exceptional stability, making it an ideal candidate for labeling applications. Based on miRFP670nano3, we established two NIR fluorescent protein labeling methods.

To insert the coding sequence of miRFP670nano3 into the genome of *Y. pestis*, we employed a CRISPR-Cas12a-assisted gene editing system (22). First, the gene encoding miRFP670nano3 was amplified by PCR from the plasmid pBR322-IP3-miRFP670nano3 (Fig. S1A). To enhance the expression of miRFP670nano3, we selected the pseudogene *yp_3493* (Fig. S1B), which exhibited a high transcriptional level in our previous study, as the insertion site. Additionally, the *ymt* promoter with high transcriptional activity (23), corresponding to the −300 to 0 bp region upstream of *ymt*, was utilized to drive expression of the miRFP670nano3 protein. Heme oxygenase-1 (HO-1) converts endogenously synthesized heme into biliverdin (24, 25), which subsequently serves as the chromophore for CBCR-derived NIR fluorescent proteins (26). To enhance biliverdin synthesis, we inserted HO-1 into the chromosome and separated it from the fluorescent protein by a self-splicing 2A peptide. Correct insertion was confirmed by PCR, and the resulting strain was named 201-nano3 (Fig. S1C). As a parallel approach, the plasmid pBR322-IP3-miRFP670nano3 was transformed into *Y. pestis* strain 201 to generate the strain 201-p-nano3.

### *In vitro* characterization of the fluorescence emission from miRFP670nano3-labeled *Y. pestis*

**Fig 1:**
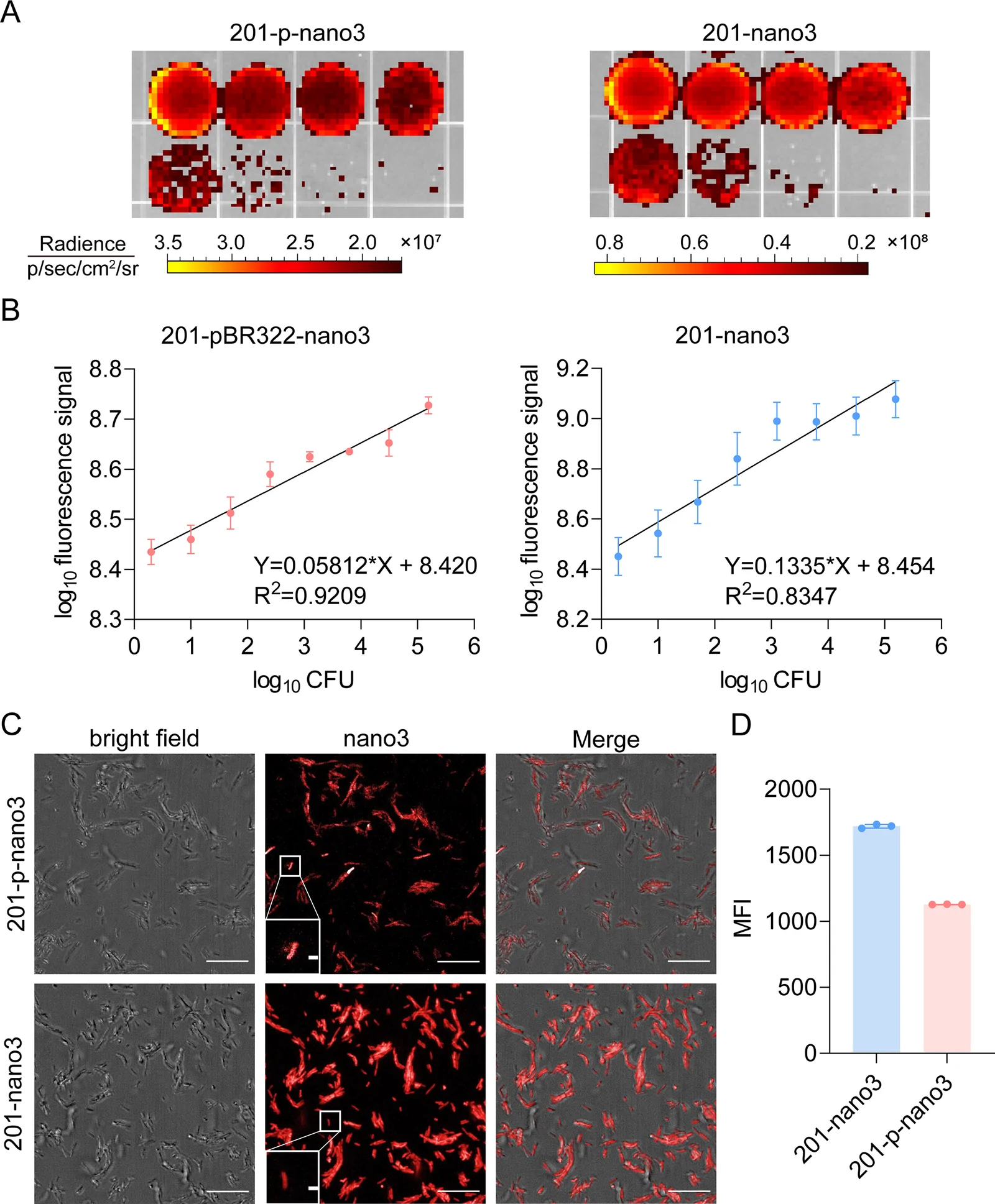
Both labeling methods enable stable NIR fluorescence emission in *Y. pestis*. (**A**) 201-p-nano3 and 201-nano3 (3.9 × 10^6^ CFU/mL) were 5-fold serially diluted and plated on Hottinger’s agar. Fluorescence of the strains was detected by IVIS. (**B**) The correlation between fluorescence intensity and bacterial number was analyzed by fitting a linear regression function, and the *R*^2^ is shown. (**C**) 201-p-nano3 and 201-nano3 were grown until an OD_620nm_ of ~1.0 and then imaged by HCI. Scale bars: 20 µm (overview) and 2 µm (magnified insets). (**D**) Mean fluorescence intensity (MFI) of NIR-FP-labeled *Y. pestis* was quantified by Harmony 5.1 over three independent experiments.

To evaluate the fluorescence signal of the NIR-FP-labeled strains, 201-p-nano3 and 201-nano3 were serially diluted by 5-fold, spotted onto Hottinger’s agar plates, cultured, and subsequently imaged using an *in vivo* imaging system (IVIS). The fluorescence intensity of 201-nano3 was higher than that of 201-p-nano3 (Fig. 1A). A strong linear correlation was observed between the bacterial numbers and the corresponding fluorescence signals following log_10_ transformation, with a detection limit of approximately 100 CFU for both strains (Fig. 1B).

Next, fluorescent imaging of both 201-p-nano3 and 201-nano3 was taken using fluorescent microscopy, and bright and uniform signals can be constantly observed at an emission wavelength of 665 nm and excitation wavelength of 650 nm. The mean fluorescence intensity (MFI) of 201-nano3 was found to be approximately 1.5-fold higher than that of 201-p-nano3. (Fig. 1C and D). Both 201-p-nano3 and 201-nano3 strains exhibited detectable NIR fluorescent signals, with a strong correlation between bacteria count and fluorescence intensity, thereby demonstrating their potential for intracellular and *in vivo* tracking applications.

### miRFP670nano3 labeling shows limited impact on the growth or virulence of *Y. pestis*

**Fig 2:**
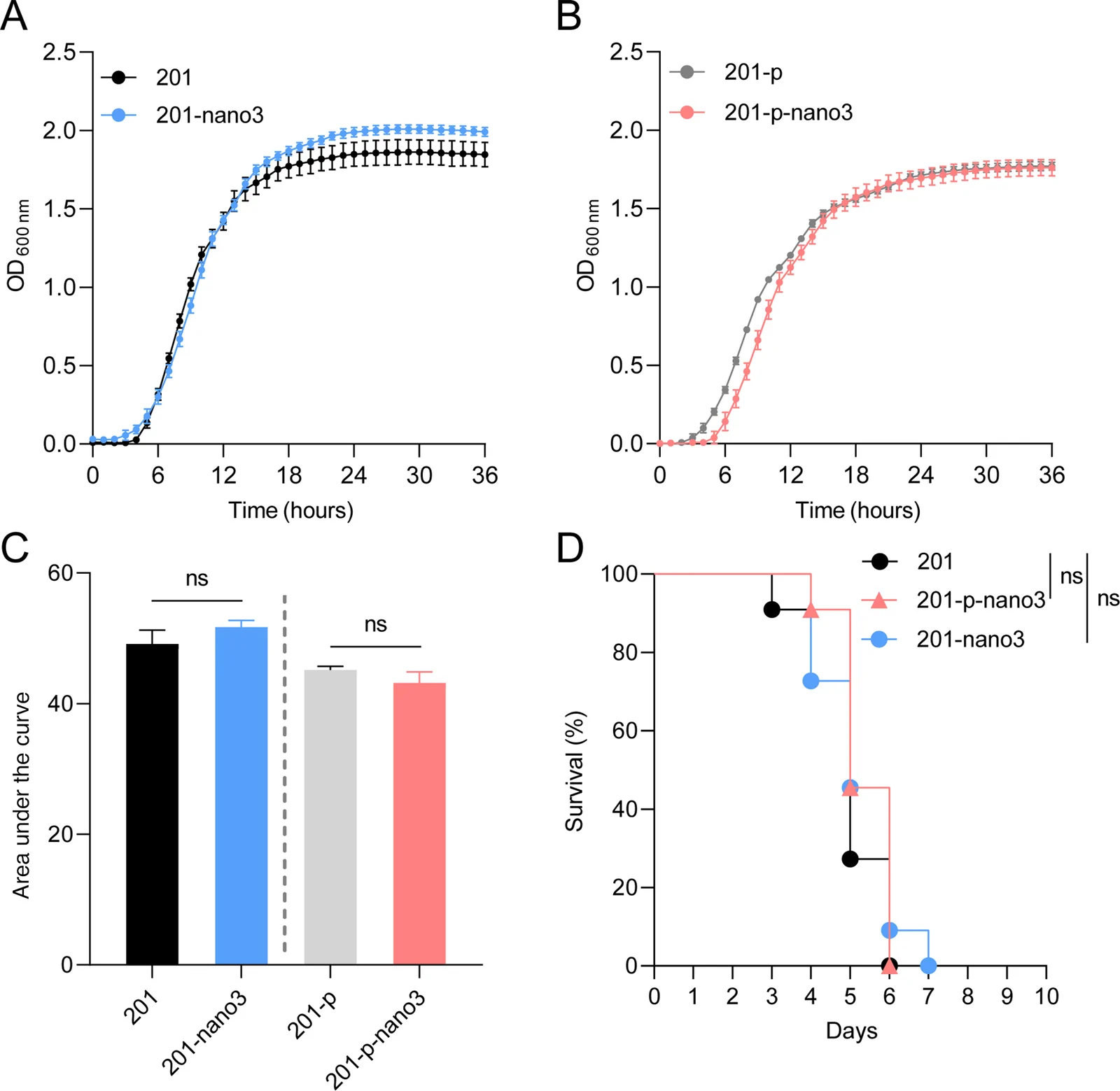
Chromosomal insertion of miRFP670nano3 or introducing its expressing plasmid does not affect bacterial growth or pathogenicity in mice. (**A and B**) *Y. pestis* strains 201, 201-nano3 (A), 201-p, and 201-p-nano3 (B) were cultured in LB medium at 26°C, with 100 µg/mL ampicillin in LB medium for 201-p and 201-p-nano3. The OD_600nm_ of each strain was recorded per hour. (**C**) The area under the growth curve was calculated, and the significance of differences within two pairs of strains was analyzed using unpaired *t*-test analysis (ns, *P* > 0.05). (**D**) BALB/c mice were infected s.c. with 1000 CFU of strain 201, 201-p-nano3, or 201-nano3 (*n* = 11 per group), and the log-rank test was used to measure the significance of differences between the survival curves (ns, *P* > 0.05).

Chromosomal insertion genes or introducing exogenous plasmids could potentially affect bacterial growth and pathogenicity. To address this concern, we compared the growth dynamics of the wild-type 201 strain, 201 carrying the empty vector, and the fluorescent strains over a 36-h period. The strains were cultured in Luria-Bertani (LB) broth at 26°C, with 100 µg/mL ampicillin in LB medium for 201-p-nano3 and 201-p carrying the empty vector. During the first 14 h, the growth curves of 201-nano3 and 201 overlapped almost entirely (Fig. 2A and B), as 201-p-nano3 exhibited slightly slower growth compared to the empty vector control. After 14 h, the growth curves of 201-p-nano3 and 201-p began to converge and became eventually indistinguishable, and 201-nano3 reached a slightly higher density at the stationary phase than 201. No significant differences in the area under the growth curve were observed within either pair of strains (Fig. 2C), indicating that the insertion of the miRFP670nano3 coding sequence or the transformation of pBR322-IP3-nano3 plasmid did not notably affect the growth of *Y. pestis*.

To assess the impact of our labeling methods on bacterial virulence, BALB/c mice were infected subcutaneously (s.c.) with approximately 1,000 CFU of either the wild-type 201 strain or the miRFP670nano3-labeled strains. No significant difference in survival curves was observed between the groups (Fig. 2D), suggesting that neither the chromosomal insertion of the miRFP670nano3 gene nor the introduction of pBR322-IP3-nano3 affected the virulence of *Y. pestis* at this dose. Therefore, these fluorescent strains are suitable for studying the dynamics of *Y. pestis* infection.

### Real-time imaging of bacteria-host interactions using miRFP670nano3-labeled *Y. pestis* strains

**Fig 3:**
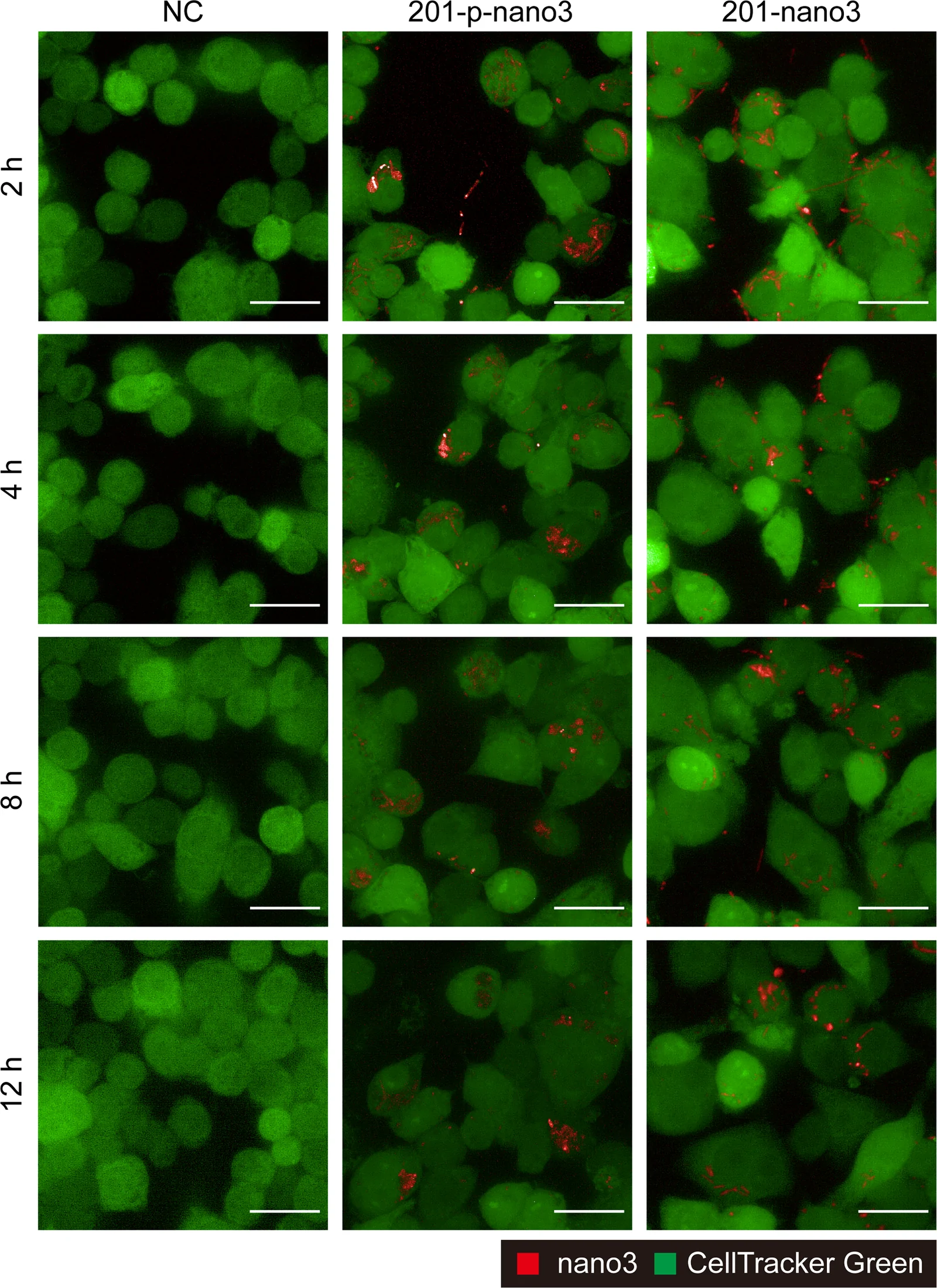
Live imaging analysis of macrophages infected with miRFP670nano3-labeled *Y. pestis*. RAW264.7 cells were stained by CellTracker Green and infected with 201-p-nano3 or 201-nano3 (MOI = 5). The dynamic interaction between macrophages and *Y. pestis* was observed for 12 h, and the images were taken at the indicated time points. Scale bar: 20 µm.

Another advantage of fluorescent labeling is that it enables the monitoring of the bacteria-cell interactions. To further evaluate the tracing capability of fluorescent strains, particularly their potential for visualizing interactions with host cells, we continuously monitored the dynamic interaction between miRFP670nano3-labeled strains and RAW264.7 cells pre-stained with CellTracker Green over a 12-h period using a High-Content Imaging and Analysis System (HCI). The fluorescent signals from the intracellular *Y. pestis* were distinct and sufficiently intense for clear observation, allowing both clumps of bacteria and individual bacteria to be visible in real time. We noted that the fluorescent signals gradually diminished over this period, suggesting that the bacteria were progressively eliminated by macrophages (Fig. 3). These results indicate that the fluorescent protein labeling system can be effectively used to study the interaction between bacteria and host cells for up to 12 h.

### *In vivo* dissemination of miRFP670nano3-labeled *Y. pestis* tracked by live imaging in a bubonic plague mouse model

**Fig 4:**
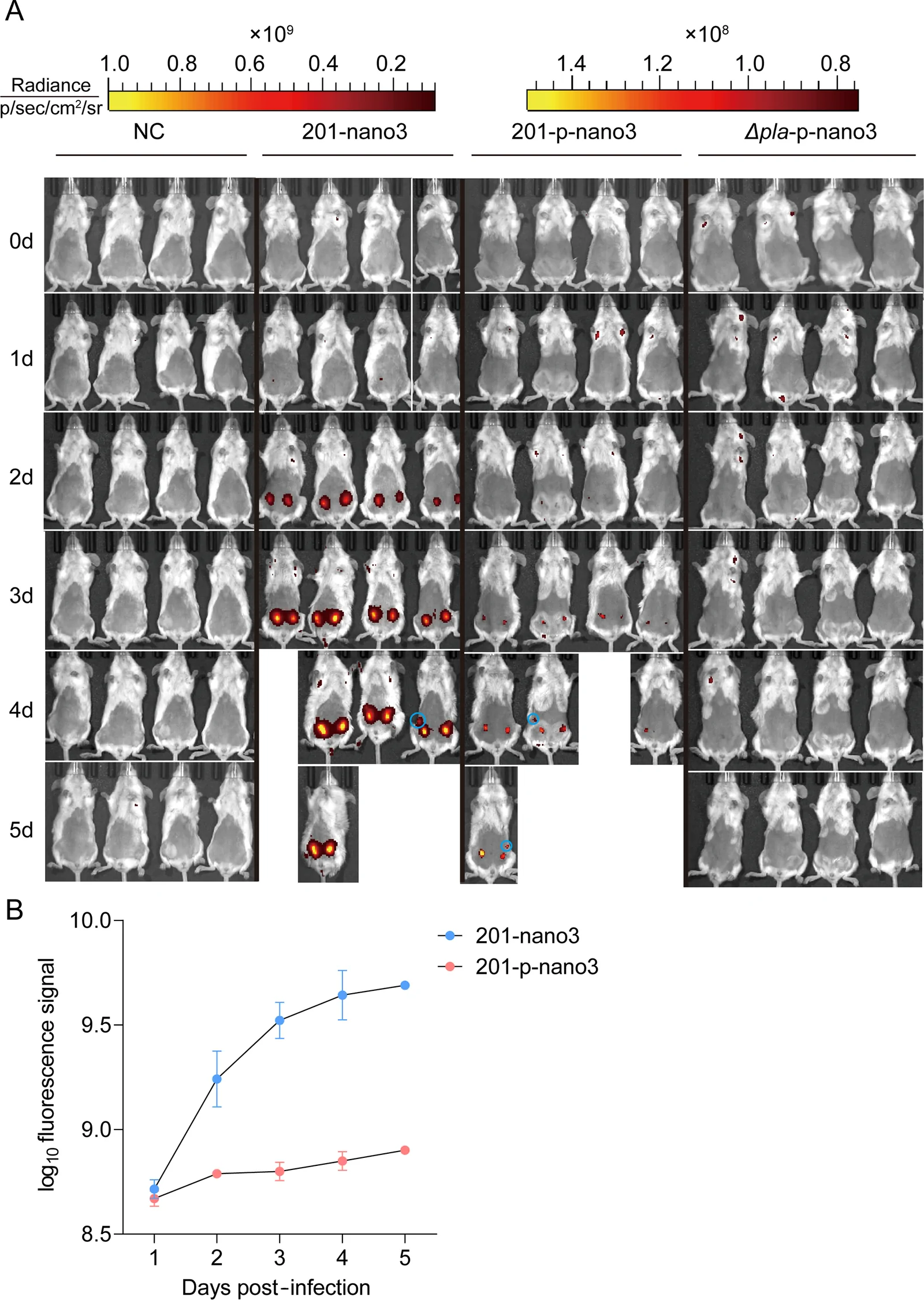
Dynamic dissemination of miRFP670nano3-labeled *Y. pestis* during the bubonic plague was monitored using IVIS analysis. (**A**) Mice (*n* = 4 per group) were s.c. challenged with approximately 1,000 CFU of the indicated bacterial strains in PBS and imaged daily by IVIS. Mice in the negative control (NC) group were injected with the same volume of PBS. Black lines separated different experimental groups, whereas white lines indicated boundaries between adjacent image segments. Blank spaces indicated mice that died before the corresponding imaging time point. (**B**) Quantification of fluorescence signal intensities in ILN is shown. Results are expressed as mean ± SDs.

Next, we assessed the ability of the NIR-FP-labeled strains to track *in vivo* infection dynamics in a murine bubonic plague model. To this end, BALB/c mice were s.c. infected with approximately 1,000 CFU of various strains and underwent daily imaging using an IVIS system. At 1 day post-infection (dpi), faint fluorescent signals gradually emerged in the inguinal lymph nodes (ILN) of mice infected with 201-nano3. The fluorescence intensity steadily increased until the mice succumbed to infection, indicating significant proliferation of *Y. pestis* in the ILN (Fig. 4A). By 4 dpi, NIR fluorescent signals were also detectable in adjacent lymph nodes (marked with blue circles), coinciding with the onset of mortality and suggesting that *Y. pestis* had established a systemic infection through the lymphatic cycle. Notably, weak fluorescent signal from mice infected with 201-nano3 was first detected at 1 dpi, whereas signals from the 201-p-nano3-infected mice remained undetectable even until 2 dpi. On the same time point, the fluorescence intensity in mice infected with 201-nano3 was markedly stronger than in those infected with 201-p-nano3. The difference in fluorescence intensity between the two strains became increasingly pronounced as the infection progressed, reaching a 7-fold increase by 3 dpi (Fig. 4B), consistent with the fluorescence microscopy and IVIS imaging results that 201-nano3 exhibited significantly brighter fluorescence than 201-p-nano3.

Then, we investigated whether this NIR-FP labeling system could be used to evaluate the virulence of different *Y. pestis* strains, particularly those defective in virulence determinants. The Pla protease plays a critical role in the dissemination of *Y. pestis* from the initial infection site to the lymphatic system and in its subsequent colonization in the lungs (27–29). Mice infected with 201-p-nano3 or Δ*pla*-p-nano3 were imaged using IVIS over 7 days of infection to compare their fluorescent signals between the two groups. Fluorescence microscopy confirmed that the fluorescence intensity of Δ*pla*-p-nano3 was comparable to that of 201-p-nano3 (Fig. S2A and B). Over the 7 days following infection (data shown up to day 5), mice infected with *Δpla*-p-nano3 showed no significant fluorescence signals in the ILN or elsewhere, unlike the strong signals observed in mice infected with either 201-p-nano3 or 201-nano3 (Fig. 4A). These findings suggest that NIR fluorescence intensity of the miRFP670nano3-labeled strains can serve as a quantifiable measure of virulence attenuations, providing a useful tool for validating the role of specific genes in bacterial transmission to the draining lymph node.

**Fig 5:**
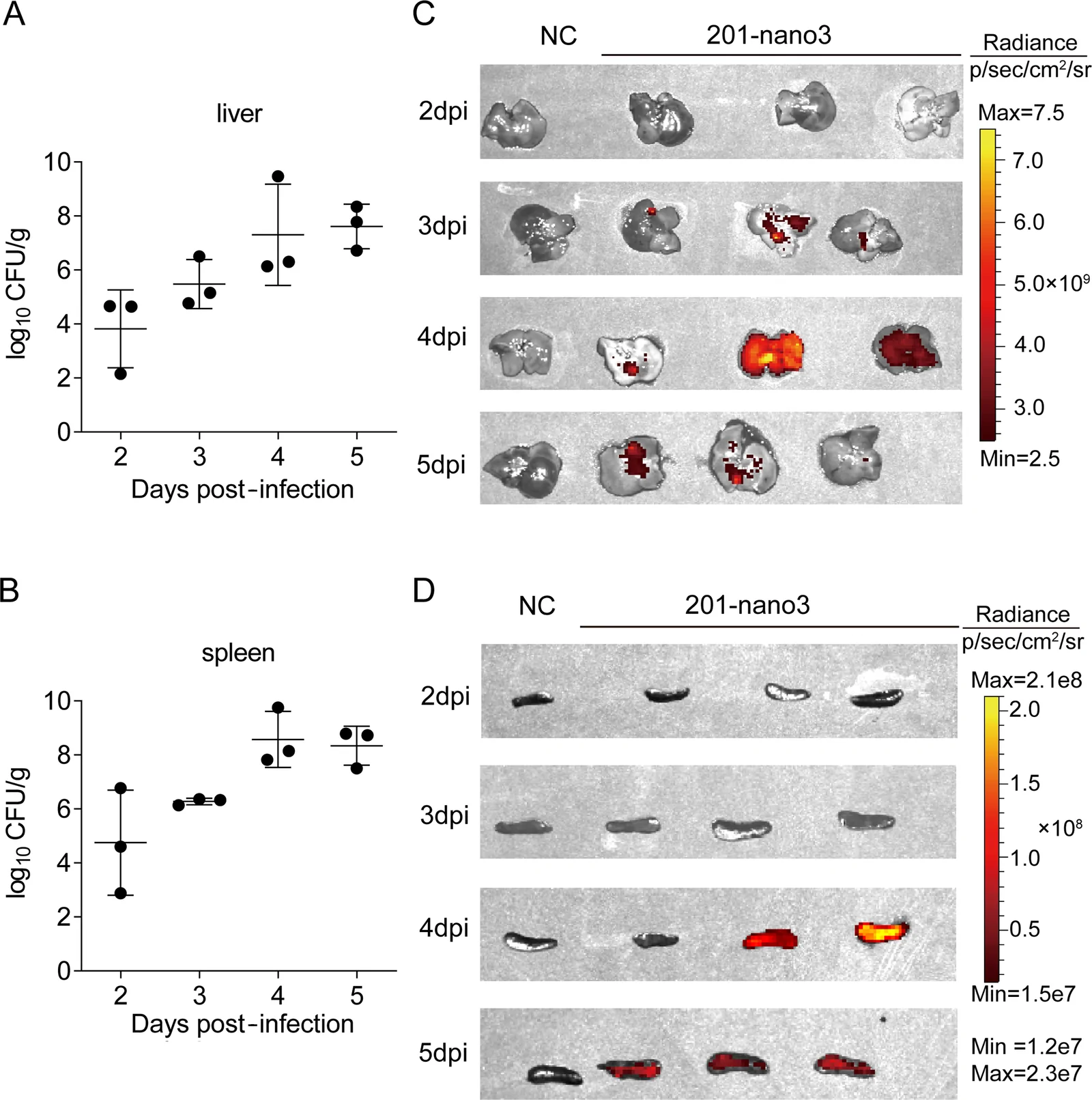
Dissemination of miRFP670nano3-labeled *Y. pestis* to deep organs was visualized *ex vivo* using IVIS. (**A and B**) Mice were challenged s.c. with 1,000 CFU of 201-nano3. Three mice per time point were sacrificed randomly from 2 to 5 dpi. Bacterial burdens in the livers (**A**) and spleens (**B**) were measured by counting living bacteria. (**C and D**) Groups of BALB/c mice were injected s.c. with PBS or 1,000 CFU 201-nano3. Three infected mice and one control mouse were euthanized daily from 2 dpi. Livers (**C**) and spleens (**D**) were collected and imaged by IVIS.

Notably, no fluorescent signal was observed in organs beyond the ILN in mice that ultimately succumbed to plague, despite the expected systemic dissemination of the bacteria. To investigate this discrepancy, we analyzed both the bacterial burden and the fluorescence intensity in organs dissected from mice infected with the 201-nano3 strain. Bacterial burdens in the livers and spleens of infected mice were analyzed from 2 to 5 dpi, peaking at approximately 10⁷ CFU/g in livers and 10⁸ CFU/g in spleens, respectively, by 4 dpi (Fig. 5A and B). Fluorescent signals were first detected at 3 dpi in livers harvested from mice infected with 201-nano3 by *ex vivo* imaging (Fig. 5C), while signals from the spleens appeared 1 day later (Fig. 5D). These results suggest that, although fluorescence from miRFP670nano3 allows detection in superficial tissues such as ILN, the limited tissue penetration of the signal makes *in vivo* imaging in deeper organs challenging with this approach.

## DISCUSSION

Live imaging enables non-invasive, real-time monitoring of pathogen infection dynamics *in vivo*, offering critical insight into infectious diseases research. Effective labeling strategies are essential to the success of *in vivo* imaging. Here, we developed two novel NIR-FP-based labeling methods to analyze the infection dynamics of *Y. pestis*. The first method introduces a plasmid expressing the miRFP670nano3 protein into *Y. pestis* strains. The second method integrates the miRFP670nano3 gene directly into the bacterial genome, providing a fluorescence-labeled strain with better stability and higher fluorescence intensity. The fluorescence of the two NIR-FP-labeled *Y. pestis* strains was demonstrated both *in vivo* and *in vitro* using IVIS and Operetta CLS imaging systems. Importantly, we confirmed that neither of the labeling methods affected the growth or virulence of the strains. Both labeling techniques enabled real-time tracking of *Y. pestis* infection dynamics in a murine bubonic plague model, representing a significant advantage for understanding the progression of infection.

Interestingly, we showed that expression of miRFP670nano3 driven by the *ymt* promoter significantly enhanced the fluorescence intensity of the labeled strains. Although the genome of 201-nano3 contained only a single copy of miRFP670nano3, its fluorescence intensity was higher than that of 201-p-nano3, which contains multiple plasmid-borne copies. These findings underscore the importance of selecting a strong promoter and a genomic insertion site with high transcriptional activity for optimal expression of fluorescent proteins, providing new insights into how targeted genetic modifications can improve fluorescence signal strength.

While the universality of these labeling methods has not been explicitly validated in other bacteria, they are promising to offer notable advantages over traditional methods. Traditionally, NIR fluorescent labeling of pathogens relies on diverse fluorescent probes conjugating dyes, such as Cy5.5 or Cy7 (30–32). However, these methods require repeated labeling of the bacteria before each infection and tracking session. The fluorescence intensity of bacteria labeled with these probes decreases as the bacteria replicate (31), making it impossible to assess bacterial burden. Additionally, fluorescent probes are limited to short-term tracking due to their rapid fluorescence decay during replication, which compromises the ability to trace the pathogen over extended periods. In contrast, the labeling methods established in this study allow for the monitoring of *Y. pestis* replication *in vivo* for several days, offering a more stable and long-term solution for dynamic infection studies.

Recently, Daiki Yamaguchi et al. (33) developed an NIR *in vivo* imaging system capable of visualizing bacteria colonization in the lungs. Their approach relies on a plasmid expressing firefly luciferase within the bacteria, which catalyzes the D-luciferin derivative “TokeOni” to emit NIR bioluminescence. Our method offers a notable advantage by enabling the observation of NIR-FP-labeled strains and their interactions with host cells without the need for substrate injection prior to imaging. Instead, we directly labeled the bacteria with an NIR protein, either by introducing a plasmid expressing it or by chromosomal insertion of its encoding gene, streamlining the procedure and improving convenience for dynamic and real-time studies.

In summary, the NIR-FP labeling methods developed in this study demonstrate significant potential for tracking *Y. pestis* infection dynamics *in vivo*, particularly in lymph nodes. However, a limitation of our approach is that fluorescence detection is primarily limited to lymph nodes, with weaker tracking capability observed in deep tissues such as the spleen and lung. The absence of detectable signals at the groin inoculation sites indicates that the inoculation amount (approximately 500 CFU per inguinal region used in this study) likely falls below the sensitivity threshold of IVIS imaging—an empirical observation in this model. Although the miRFP670nano3 NIR fluorophores offer improved tissue penetrance over visible-light reporters, the detection limit remains constrained by photon absorption, scattering, and signal attenuation through deep anatomical structures. To address this, the development of brighter NIR fluorescent proteins with deeper tissue penetration would further enhance the sensitivity and accuracy of live imaging, allowing for more comprehensive monitoring of pathogen dynamics across various organ systems.

## MATERIALS AND METHODS

### Bacterial strains and culture conditions

The bacterial strains and plasmids used in this study were listed in Table S1.

Strains were grown in Lysogeny broth (LB) broth or on Hottinger’s agar plate at 26°C. When needed, ampicillin was added at 100 μg/mL and chloramphenicol at 34 μg/mL. The *Y. pestis* 201 strain is avirulent to humans (34). All culture and infection experiments involving *Y. pestis* 201 strain were conducted in a biosafety level-2 laboratory.

### Cell cultures

RAW264.7 cells were cultured in DMEM medium containing 10% fetal bovine serum (Gibco, USA) at 37°C in a 5% CO2_2_ incubator.

### Construction of near-infrared fluorescent protein-labeled *Y. pestis*

*Y. pestis* wild-type strain 201 belonging to the biovar Microtus was used in this study (34). 201-nano3 was constructed based on the CRISPR-Cas12a-assisted gene editing system. The miRFP670nano3-encoding gene and HO-1 sequence were inserted into a highly transcribed pseudogene locus *yp_3493*. To further improve the expression of miRFP670nano3, the *ymt* promoter was inserted right upstream of the *miRFP670nano3* gene. Homologous arms, the *ymt* promoter, and nano3 gene were assembled by fusion PCR, and the resulting PCR products were purified using a Gel Extraction Kit. *Y. pestis* 201 carrying pKD46-FnCpf1 was electroporated with a mixture of the purified PCR products and crRNA-expressing plasmid pYC1000-eforRED-SacB, plated onto LB agar supplemented with ampicillin and chloramphenicol, and cultured overnight at 26°C. The insertion of the target fragment into the *Y. pestis* chromosome was verified by PCR amplification and sequencing. Primers used for construction of 201-nano3 were listed in Table S2. The plasmids pYC1000-eforRED-SacB and pKD46-FnCpf1 were cured by incubating the bacteria in LB broth supplemented with 7% sucrose or culturing at 42°C successively.

The empty plasmid pBR322 was transformed into strain 201, and pBR322-IP3-miRFP670nano3 plasmid was transformed into strain 201 or *Δpla* by electroporation to obtain 201-p, 201-p-nano3, and *Δpla*-p-nano3, respectively.

### Fluorescent strains imaging *in vitro*

*Y. pestis* strains 201-p-nano3 and 201-nano3 (3.9 × 10^6^ CFU/mL) were 5-fold serially diluted, and 40 µL of bacterial suspensions were spotted on the Hottinger’s agar. After culturing at 26°C for 24 h, bacterial lawns on the plates were imaged using IVIS (PerkinElmer, USA). ROI (region of interest) was defined, and the total signal of the selected region was calculated in Living Image (v.4.4, Caliper LifeSciences). The correlation between the numbers of bacteria and fluorescence signal intensities was analyzed based on simple linear regression using GraphPad Prism (v.9.5.0, GraphPad Software).

An aliquot of 1 mL bacterial suspensions of 201-p-nano3, 201-nano3, or *Δpla-*p-nano3 at OD_620nm_=1.0 was centrifuged at 2,000 × *g*, and the supernatants were discarded. The pellets were then resuspended in 1 mL PBS, followed by serial 10-fold dilutions. Fifty microliters of the diluted bacterial suspensions were added to a 96-well plate. After allowing the suspensions to settle for 5 min, the samples were imaged using an Operetta CLS High-Content Imaging and Analysis System (PerkinElmer, USA).

### Growth curve determination of fluorescent protein-labeled *Y. pestis* strains

*Y. pestis* strains 201, 201-p-nano3, 201-p, and 201-nano3 were grown at 26°C in LB medium until OD_620nm_ of ~1.0. Then, bacterial cultures were diluted 1:100 into 60 mL LB medium and incubated continuously at 26°C in Growth Curve Online Monitoring Shaker ZWYF-290 (Zhi China). Bacterial growth was monitored per hour by measuring OD_600 nm_ for 36 h.

### Assessment of the *Y. pestis* virulence in mice

Female 6–8 -week-old BALB/c mice were purchased from the Vitalriver Company (Beijing, China). *Y. pestis* strains 201, 201-p-nano3, and 201-nano3 were grown at 26°C in LB medium until OD_620nm_ of ~1.0. Mice were s.c. challenged with approximately 1,000 CFU of bacteria in a PBS suspension at the inguinal region. Ampicillin was intraperitoneally (i.p.) injected at 100 mg/kg twice daily into mice infected with 201-p-nano3 to ensure the presence of the plasmid expressing miRFP670nano3. Mice were monitored once daily for mortality.

### Bacterial dissemination assay

BALB/c mice were s.c. infected with approximately 1,000 CFU of 201-nano3 at the inguinal region. Every 24 h after infection, three mice were euthanized randomly, and their organs were collected into tubes containing 500 μL of PBS. The organs were then homogenized at 70 Hz for 3 min using a tissue grinder, Scientz-48L (SCIENTZ, China). The homogenates were serially diluted and then plated on Hottinger’s agar and incubated at 26°C. After 48 h, the number of bacteria on plates was counted.

### *In vivo* fluorescent imaging

*Y. pestis* strains 201, 201-p-nano3, 201-nano3, and Δ*pla-*p-nano3 were grown at 26°C in LB medium until an OD_620nm_ of ~1.0. Mice were s.c. infected with 1,000 CFU bacteria at the inguinal region. Ampicillin was i.p. injected at 100 mg/kg twice daily into mice infected with strains carrying the miRFP670nano3-expressing plasmid. Mice were anesthetized with 1.25% isoflurane during imaging to maintain a stable position and monitored by IVIS once daily. Imaging parameters were set as follows: fluorescence mode, exposure auto, binning medium, F-stop 8, excitation 640 nm, emission 680 nm, FOV D, and height 1.5. To quantify the fluorescence intensity from strains in a specific organ, we used ROI tools to define the “region of interest’’ and calculated the total signal of the selected region.

## Data Availability

All data generated during this study are included in this article and its supplemental materials. The plasmid pBR322-IP3-miRFP670nano3 used in this study has been deposited in plasmid repository WeKwikGene (https://wekwikgene.wllsb.edu.cn) under barcode 0003057.

## SUPPLEMENTAL MATERIAL

Supplemental material spectrum.00959-26-s0001.docx Tables S1 and S2 and Fig. S1 and S2.

ASM does not own the copyrights to Supplemental Material that may be linked to, or accessed through, an article. The authors have granted ASM a non-exclusive, world-wide license to publish the Supplemental Material files. Please contact the corresponding author directly for reuse.

## References

[1] St John AL, Ang WXG, Huang M-N, Kunder CA, Chan EW, Gunn MD, Abraham SN. 2014. S1P-Dependent trafficking of intracellular yersinia pestis through lymph nodes establishes Buboes and systemic infection. Immunity 41:440–450. doi:10.1016/j.immuni.2014.07.013 PMC4440548 25238098

[2] Gonzalez RJ, Lane MC, Wagner NJ, Weening EH, Miller VL. 2015. Dissemination of a highly virulent pathogen: tracking the early events that define infection. PLoS Pathog 11:e1004587. doi:10.1371/journal.ppat.1004587 PMC4303270 25611317

[3] Kamenyeva O, Boularan C, Kabat J, Cheung GYC, Cicala C, Yeh AJ, Chan JL, Periasamy S, Otto M, Kehrl JH. 2015. Neutrophil recruitment to lymph nodes limits local humoral response to *Staphylococcus aureus**.* PLoS Pathog 11:e1004827. doi:10.1371/journal.ppat.1004827 PMC4401519 25884622

[4] Xue J, Lin Y, Oo D, Zhang J, Zardynezhad A, de Jesus FN, Stephens M, de Almeida LGN, Young D, Dufour A, Liao S. 2022. Lymph-derived chemokines direct early neutrophil infiltration in the lymph nodes upon *Staphylococcus aureus* skin infection. Proc Natl Acad Sci USA 119:e2111726119. doi:10.1073/pnas.2111726119 PMC9371737 35914162

[5] Lynskey NN, Banerji S, Johnson LA, Holder KA, Reglinski M, Wing PAC, Rigby D, Jackson DG, Sriskandan S. 2015. Rapid lymphatic dissemination of encapsulated group a streptococci via lymphatic vessel endothelial receptor-1 interaction. PLoS Pathog 11:e1005137. doi:10.1371/journal.ppat.1005137 PMC4564194 26352587

[6] Siggins MK, Lynskey NN, Lamb LE, Johnson LA, Huse KK, Pearson M, Banerji S, Turner CE, Woollard K, Jackson DG, Sriskandan S. 2020. Extracellular bacterial lymphatic metastasis drives *Streptococcus pyogenes* systemic infection. Nat Commun 11:4697. doi:10.1038/s41467-020-18454-0 PMC7498588 32943639

[7] Bosio CF, Jarrett CO, Gardner D, Hinnebusch BJ. 2012. Kinetics of innate immune response to *Yersinia pestis* after intradermal infection in a mouse model. Infect Immun 80:4034–4045. doi:10.1128/IAI.00606-12 PMC3486050 22966041

[8] Demeure CE, Blanchet C, Fitting C, Fayolle C, Khun H, Szatanik M, Milon G, Panthier JJ, Jaubert J, Montagutelli X, Huerre M, Cavaillon JM, Carniel E. 2012. Early systemic bacterial dissemination and a rapid innate immune response characterize genetic resistance to plague of SEG mice. J Infect Dis 205:134–143. doi:10.1093/infdis/jir696 22090450

[9] Gonzalez RJ, Weening EH, Frothingham R, Sempowski GD, Miller VL. 2012. Bioluminescence imaging to track bacterial dissemination of Yersinia pestis using different routes of infection in mice. BMC Microbiol 12:147. doi:10.1186/1471-2180-12-147 PMC3436865 22827851

[10] Nham T, Filali S, Danne C, Derbise A, Carniel E. 2012. Imaging of bubonic plague dynamics by *in vivo* tracking of bioluminescent *Yersinia pestis*. PLoS One 7:e34714. doi:10.1371/journal.pone.0034714 PMC3320629 22496846

[11] Bosio CF, Jarrett CO, Scott DP, Fintzi J, Hinnebusch BJ. 2020. Comparison of the transmission efficiency and plague progression dynamics associated with two mechanisms by which fleas transmit *Yersinia pestis*. PLoS Pathog 16:e1009092. doi:10.1371/journal.ppat.1009092 PMC7746306 33284863

[12] Andreu N, Zelmer A, Wiles S. 2011. Noninvasive biophotonic imaging for studies of infectious disease. FEMS Microbiol Rev 35:360–394. doi:10.1111/j.1574-6976.2010.00252.x PMC3084502 20955395

[13] Jiang T, Bai X, Li M. 2024. Advances in the development of bacterial bioluminescence imaging. Annu Rev Anal Chem (Palo Alto Calif) 17:265–288. doi:10.1146/annurev-anchem-061622-034229 38640069

[14] Rizzo MA, Davidson MW, Piston DW. 2009. Fluorescent protein tracking and detection: applications using fluorescent proteins in living cells. Cold Spring Harb Protoc 2009:pdb.top64. doi:10.1101/pdb.top64 20150101

[15] Enterina JR, Wu L, Campbell RE. 2015. Emerging fluorescent protein technologies. Curr Opin Chem Biol 27:10–17. doi:10.1016/j.cbpa.2015.05.001 26043278

[16] van Zyl WF, Deane SM, Dicks LMT. 2015. Use of the mCherry fluorescent protein to study intestinal colonization by *Enterococcus mundtii* ST4SA and *Lactobacillus plantarum* 423 in mice. Appl Environ Microbiol 81:5993–6002. doi:10.1128/AEM.01247-15 PMC4551250 26116681

[17] Nogales A, Baker SF, Martínez-Sobrido L. 2015. Replication-competent influenza A viruses expressing a red fluorescent protein. Virology 476:206–216. doi:10.1016/j.virol.2014.12.006 PMC4323957 25553516

[18] Shu X, Royant A, Lin MZ, Aguilera TA, Lev-Ram V, Steinbach PA, Tsien RY. 2009. Mammalian expression of infrared fluorescent proteins engineered from a bacterial phytochrome. Science 324:804–807. doi:10.1126/science.1168683 PMC2763207 19423828

[19] Filonov GS, Piatkevich KD, Ting LM, Zhang J, Kim K, Verkhusha VV. 2011. Bright and stable near-infrared fluorescent protein for *in vivo* imaging. Nat Biotechnol 29:757–761. doi:10.1038/nbt.1918 PMC3152693 21765402

[20] Shcherbakova DM, Verkhusha VV. 2013. Near-infrared fluorescent proteins for multicolor *in vivo* imaging. Nat Methods 10:751–754. doi:10.1038/nmeth.2521 PMC3737237 23770755

[21] Oliinyk OS, Baloban M, Clark CL, Carey E, Pletnev S, Nimmerjahn A, Verkhusha VV. 2022. Single-domain near-infrared protein provides a scaffold for antigen-dependent fluorescent nanobodies. Nat Methods 19:740–750. doi:10.1038/s41592-022-01467-6 PMC9189029 35606446

[22] Yan MY, Yan HQ, Ren GX, Zhao JP, Guo XP, Sun YC. 2017. CRISPR-Cas12a-assisted recombineering in bacteria. Appl Environ Microbiol 83:e00947-17. doi:10.1128/AEM.00947-17 PMC5561284 28646112

[23] Chen H, Cao S, Zhou Y, Wang T, Jiao Y, Tan Y, Wu Y, Ren Y, Song Y, Zhang J-R, Du Z, Yang R. 2025. Molecular turn in *Yersinia pestis* pathogenesis: implications of the *gppA* frameshift for bacterial survival in human macrophage. Emerg Microbes Infect 14:2467778. doi:10.1080/22221751.2025.2467778 PMC11878169 39945756

[24] Rodriguez EA, Tran GN, Gross LA, Crisp JL, Shu X, Lin JY, Tsien RY. 2016. A far-red fluorescent protein evolved from a cyanobacterial phycobiliprotein. Nat Methods 13:763–769. doi:10.1038/nmeth.3935 PMC5007177 27479328

[25] Shemetov AA, Oliinyk OS, Verkhusha VV. 2017. How to increase brightness of near-infrared fluorescent proteins in mammalian cells. Cell Chem Biol 24:758–766. doi:10.1016/j.chembiol.2017.05.018 PMC5519290 28602760

[26] Oliinyk OS, Shemetov AA, Pletnev S, Shcherbakova DM, Verkhusha VV. 2019. Smallest near-infrared fluorescent protein evolved from cyanobacteriochrome as versatile tag for spectral multiplexing. Nat Commun 10:279. doi:10.1038/s41467-018-08050-8 PMC6336887 30655515

[27] Wu G, Zhou Y, Cao S, Wu Y, Wang T, Zhang Y, Wang X, Song Y, Yang R, Du Z. 2024. Unveiling the dance of evolution: Pla-mediated cleavage of Ymt modulates the virulence dynamics of *Yersinia pestis*. mBio 15:e0107524. doi:10.1128/mbio.01075-24 PMC11323527 38958447

[28] Guinet F, Avé P, Filali S, Huon C, Savin C, Huerre M, Fiette L, Carniel E. 2015. Dissociation of tissue destruction and bacterial expansion during bubonic plague. PLoS Pathog 11:e1005222. doi:10.1371/journal.ppat.1005222 PMC4615631 26484539

[29] Sebbane F, Jarrett CO, Gardner D, Long D, Hinnebusch BJ. 2006. Role of the *Yersinia pestis* plasminogen activator in the incidence of distinct septicemic and bubonic forms of flea-borne plague. Proc Natl Acad Sci USA 103:5526–5530. doi:10.1073/pnas.0509544103 PMC1414629 16567636

[30] Ferreira K, Hu H-Y, Fetz V, Prochnow H, Rais B, Müller PP, Brönstrup M. 2017. Multivalent siderophore-DOTAM conjugates as theranostics for imaging and treatment of bacterial infections. Angew Chem Int Ed Engl 56:8272–8276. doi:10.1002/anie.201701358 28608939

[31] Liu F, Chen S, Zou Y, Jiao Y, Tang Y. 2023. A simple and efficient fluorescent labeling method in *Staphylococcus aureus* for real-time tracking of invasive bacteria. Front Microbiol 14:1128638. doi:10.3389/fmicb.2023.1128638 PMC9950555 36846783

[32] Li Y, Zhou Y, Du Y, Gao P, Yang L, Wang W. 2024. *In vivo* labeling and intravital imaging of bacterial infection using a near-infrared fluorescent D-amino acid probe. Chembiochem 25:e202400283. doi:10.1002/cbic.202400283 38715148

[33] Yamaguchi D, Kamoshida G, Kawakubo S, Azuma S, Tsuji T, Kitada N, Saito-Moriya R, Yamada N, Tanaka R, Okuda A, Ueyama K, Isaka S, Tomita M, Nakano R, Morita Y, Yano H, Maki SA, Yahiro K, Kato S. 2024. Near-infrared *in vivo* imaging system for dynamic visualization of lung-colonizing bacteria in mouse pneumonia. Microbiol Spectr 12:e0082824. doi:10.1128/spectrum.00828-24 PMC11537041 39287455

[34] Song Y, Tong Z, Wang J, Wang L, Guo Z, Han Y, Zhang J, Pei D, Zhou D, Qin H, et al.. 2004. Complete genome sequence of *Yersinia pestis* strain 91001, an isolate avirulent to humans. DNA Res 11:179–197. doi:10.1093/dnares/11.3.179 15368893

