# Supplementary material for: Real-time tracking of Yersinia pestis infection dynamics using a near-infrared fluorescent protein labeling system
Source: Microbiol Spectr. 2026 Sep 10;14(10):e00959-26. doi: 10.1128/spectrum.00959-26 (PMC13689436; doi:10.1128/spectrum.00959-26)
Supplement: Supplementary file 1 [file spectrum.00959-26-s0001.docx]

**Supplementary Table 1. Strains and plasmids used in this study**

| **Strains or plasmid** | **descriptions** | **sources** |
| --- | --- | --- |
| **Strains** |  |  |
| 201 | Wild type *Y. pestis* biovar Microtus strain | 34 |
| *Δpla* | 201 with deletion of *pla* using CRISPR-Cas12a-assisted recombineering system | 27 |
| 201-p | 201 carrying plasmid pBR322 | this study |
| 201-p-nano3 | 201 carrying plasmid pBR322-IP3-miRFP670nano3 | this study |
| 201-nano3 | 201 with insertion of nano3-encoding gene using CRISPR-Cas12a-assisted recombineering system | this study |
| *Δpla*-p-nano3 | *Δpla* carrying plasmid pBR322-IP3-miRFP670nano3 | this study |
| **Plasmids** |  |  |
| pBR322-IP3-miRFP670nano3 | Plasmid with near-infrared protein miRFP670nano3, Ampr | gift from Dr. Dianbing Wang |
| pKD46-FnCpf1 | pKD46 inserted with the FnCpf1 open reading frame (ORF) sequence, Ampr | 22 |
| pYC1000-eforRED SacB | Sucrose-sensitive plasmid used for inserting 25-bp targeting sequence for specific genes, Cmr | 23 |

**Supplementary Table 2. Primers used in this study**

| **Primers** | **Sequence (5’-3’)** | **Purpose** |
| --- | --- | --- |
| Homo-L1 | CAGATCGCAATGCGGCTATTGCTTATCTAATG | amplify upstream homologous arm and *ymt* promoter |
| ymtp | CTTGTCCAGGTTTGCCATATTTTATCCTTTGTTTATCGATG |  |
| nano3H-1 | CAAAGGATAAAATATGGCAAACCTGGACAAGATGCTGAAC | amplify *miRFP670nano3* gene |
| nano3H-2 | GAATATCCCCCCCCCTTTTATCACATAGCATAGAGCCCCA |  |
| Homo-R1 | CTCTATGCTATGTGATAAAAGGGGGGGGGATATTC | amplify downstream homologous arm |
| Homo-R2 | CAGCCATTTCGTTTCCATTTGGC |  |
| nano3-F | CTCTGATGATCACTAGCGGCATG | verify the successful insertion of the target fragment |
| nano3-R | GAATCCCAGCAACTGCTCTATTG |  |

**Fig S1 Construction of miRFP670nano3-labeled *Y. pestis*.**

(A) Map of plasmid pBR322-IP3-miRFP670nano3. (B) Schematic representation of insertion of miRFP670nano3 into Y. pestis chromosome for 201-nano3 construction. (C) The correct insertion of miRFP670nano3 was confirmed by PCR. The primers binding sites for PCR validation are indicated with red arrows. Lanes 1~3 correspond to the constructed strain 201-nano3, the wild type 201 and blank control, respectively.

**Fig S2 *In vitro* characterization of the fluorescence emission from *Δpla*-p-nano3.**

(A) *In vitro* imaging of strains 201-p-nano3 and *Δpla-*p-nano3 by HIC. The scale bars represent 20 µm. (B) Quantification of the MFI values for strains 201-p-nano3 and Δ*pla-*p-nano3 were analyzed by Harmony5.1.
